# Randomised controlled trial of music listening combined with progressive muscle relaxation for mood management in women receiving chemotherapy for cancer

**DOI:** 10.1007/s00520-025-09281-4

**Published:** 2025-03-04

**Authors:** Khanh Thi Nguyen, Dorothy N. S. Chan, Ankie Tan Cheung, Huong Thi Xuan Hoang, Anh Tuan Truong, Ka Ming Chow, Kai Chow Choi, Carmen W. H. Chan

**Affiliations:** 1 The Nethersole School of Nursing, The Chinese University of Hong Kong, Hong Kong SAR, China; 2 Faculty of Nursing, Phenikaa University, Hanoi, Vietnam; 3 Nam Dinh University of Nursing, Nam Dinh, Vietnam

**Keywords:** Music listening, Autogenic training, Mood, Psychological distress, Neoplasms, Antineoplastic agents

## Abstract

**Aims:**

To evaluate the effects of passive music listening combined with progressive muscle relaxation on anxiety, depression, stress, coping, and quality-of-life in women with breast and gynaecological cancers receiving chemotherapy.

**Methods:**

This was an assessor-blinded, randomised wait-list controlled trial. A total of 120 participants were randomly allocated into an intervention group or a wait-list control group. The intervention group received an intervention comprising training on passive music listening and progressive muscle relaxation, with once-daily self-practice at home for 3 weeks. The wait-list control group received the same intervention after the outcome assessment at week 6. All outcome data were collected before (T0) and 3 weeks (T1), 6 weeks (T2), and 12 months (T3) after randomisation. A generalised estimating equations model was used to compare the changes in each outcome at different time points. Process evaluation was conducted using data from the patient’s self-report forms and interviews.

**Results:**

The findings indicated that at T1 and T2, the intervention group’s reductions in anxiety were significantly larger than those of the control group. Additionally, the intervention group exhibited significantly better decreases in depression at T2, stress at T1, and dysfunctional coping at T2, and a greater improvement in quality-of-life score at T1 and T2 when compared to the control group. Most of the interviewed participants provided positive feedback on the intervention.

**Conclusions:**

The intervention was beneficial for lowering anxiety, depression, and stress and increasing the quality-of-life of women receiving chemotherapy for breast and gynaecological cancers.

Trial registration.

The trial was prospectively registered with ClinicalTrials.gov on 9 February 2022 (registration number: NCT05262621).

**Supplementary Information:**

The online version contains supplementary material available at 10.1007/s00520-025-09281-4.

## Introduction

Breast and gynaecological cancers are the most prevalent types of cancer affecting women worldwide. In 2020, approximately 3.7 million new cases of breast and gynaecological cancers were reported, accounting for approximately 40% of all incident cancers among women worldwide [50]. Compared to women with other types of cancers, those with breast and gynaecological cancers may have additional negative mood states with changes in body image and sexual health. Moreover, a majority of women with breast and gynaecological cancers are treated with chemotherapy to inhibit cancer cell proliferation and tumour multiplication [25, 37]. Although chemotherapy undeniably benefits patients in terms of improving clinical outcomes, it also causes a wide range of adverse effects, such as fatigue, neurological effects, and nausea, which have detrimental effects on the mood disturbance of patients. Around 26.9% of patients with breast cancer who undergo chemotherapy encounter anxiety, while approximately 41.55% of them face depression [49], and these rates are much higher among patients with gynaecological cancers (e.g., cervical cancer), at 52.2% and 65.6%, respectively [53].

According to the transactional model of stress and coping [31], problem- and/or emotion-focused coping strategies were chosen to handle a stressful situation. Problem-focused coping is a process focused on altering the problem; it is an attempt to directly address the source of the stress, while emotion-focused coping focuses on managing emotion when the problem cannot be changed [31]. As a form of functional emotion-focused coping, music intervention has evolved to address the growing need to provide effective stress management therapy to cancer patients receiving chemotherapy [41].

Music interventions can be classified as either music therapy or music medicine, based on the qualifications of the interventionist and the nature of the therapeutic relationship established [7]. In music therapy, a therapeutic relationship between trained music therapists and the clients is essential, enabling personally tailoring various strategies, such as active music listening, songwriting, and lyric analysis to improve health outcomes [7]. In contrast, music medicine typically involves passive music listening, where health professionals deliver pre-recorded music to patients without a structured therapeutic process [7]. Although previous studies have emphasised the importance of the therapist’s role and the therapeutic relationship [35, 44], recent evidence has found no significant difference in the effectiveness of music therapy versus music medicine in reducing anxiety among cancer patients undergoing chemotherapy [41]. Research on passive music listening has identified an effective approach for relieving stress-related anxiety among breast cancer patients undergoing chemotherapy [10, 33].

Previous studies have shown a potential increased effect of music intervention on mood management when combined with other non-pharmacological interventions, such as progressive muscle relaxation [32, 54], mindfulness-based stress reduction [34], and imagery [11–13, 45]. In the above-mentioned studies, music intervention combined with progressive muscle relaxation used passive music listening (music medicine), while other combined interventions employed music therapy. Passive music listening combined with progressive muscle relaxation (PML-PMR) was found to significantly impact anxiety and depression in female patients with breast cancer after radical mastectomy [54]. Moreover, PML-PMR led to a greater reduction in depression levels among cancer patients, compared to progressive muscle relaxation alone [32].

Although some successful stress management programmes use either passive music listening or progressive muscle relaxation, no previous studies focused on PML-PMR have involved patients with breast and gynaecological cancers who are receiving chemotherapy. The objectives of this study are (1) to examine the effects of PML-PMR on patients’ anxiety, depression, stress, coping, and quality-of-life and (2) to evaluate the implementation process to provide information needed for further research and practice.

## Methods

### Study design

This was a two-arm assessor-blinded, randomised wait-list controlled trial. A repeated-measures design was adopted, and the outcomes were assessed at four-time points: baseline (T0), immediately post-intervention (week 3, T1), 6-week follow-up (T2), and 12-month follow-up (T3).

### Settings, participants, and sample size

Participants were recruited from the Hanoi Oncology Hospital between April and August 2022 and Nam Dinh General Hospital in Vietnam between September and October 2023. Women who met the following criteria were included: a diagnosis of breast or gynaecological cancer; 18 years of age or older; a patient’s caregiver willing to support the study programme; at least 6 weeks of chemotherapy treatments remaining; a Karnofsky score [38] ≥ 80; an anxiety score > 7 or depression score > 9 (cut-off points based on the Depression Anxiety Stress Scales [DASS-21]); ability to communicate, read and write in Vietnamese; possession of a device (e.g., smartphone or MP3 player) to play the audio file and consent to join the study. Patients who met the following criteria were excluded: known psychiatric morbidity that might prevent them from following the intervention and data collection instructions; difficulty or inability to practise progressive muscle relaxation or listen to music; use of other treatments or therapies (e.g., yoga or meditation) to manage negative mood states; and personal plans or possible treatments that would hinder them from practising daily during the intervention period.

The sample size calculation was based on effect sizes of 1.2 and 0.76, respectively, for anxiety and depression and an anticipated attrition rate of 17%, which were estimated from our pilot study of women with breast or gynaecological cancer who had mild or more severe anxiety and depression at baseline. We estimated that a sample size of 45 per group would give the study 80% power, at a 5% level of significance (two-sided), to detect a conservative effect size of at least 0.6 for the primary outcomes of anxiety and depression after the intervention. After allowing for an attrition rate of 20% and rounding up the sample size, we aimed to recruit 120 participants (*n* = 60 per group).

### Recruitment, randomisation, and blinding

Potential participants were identified from medical records by four clinical nurses and a research nurse. The program information was provided before the nurses asked the patients to provide written informed consent and complete the baseline assessment if they agreed to participate.

Randomisation was performed by an independent researcher using an online randomiser to assign the participants at a 1:1 ratio to either the intervention or the wait-list control group. Block randomisation procedures with a random mix of block sizes (4, 6, and 8) were used [19]. Sequential numbered opaque envelopes were used to seal the randomisation sequence. The participants and interveners could not be blinded because of the nature of the study [27]. However, the outcome assessors were blinded.

### Study intervention

**Fig. 1:**
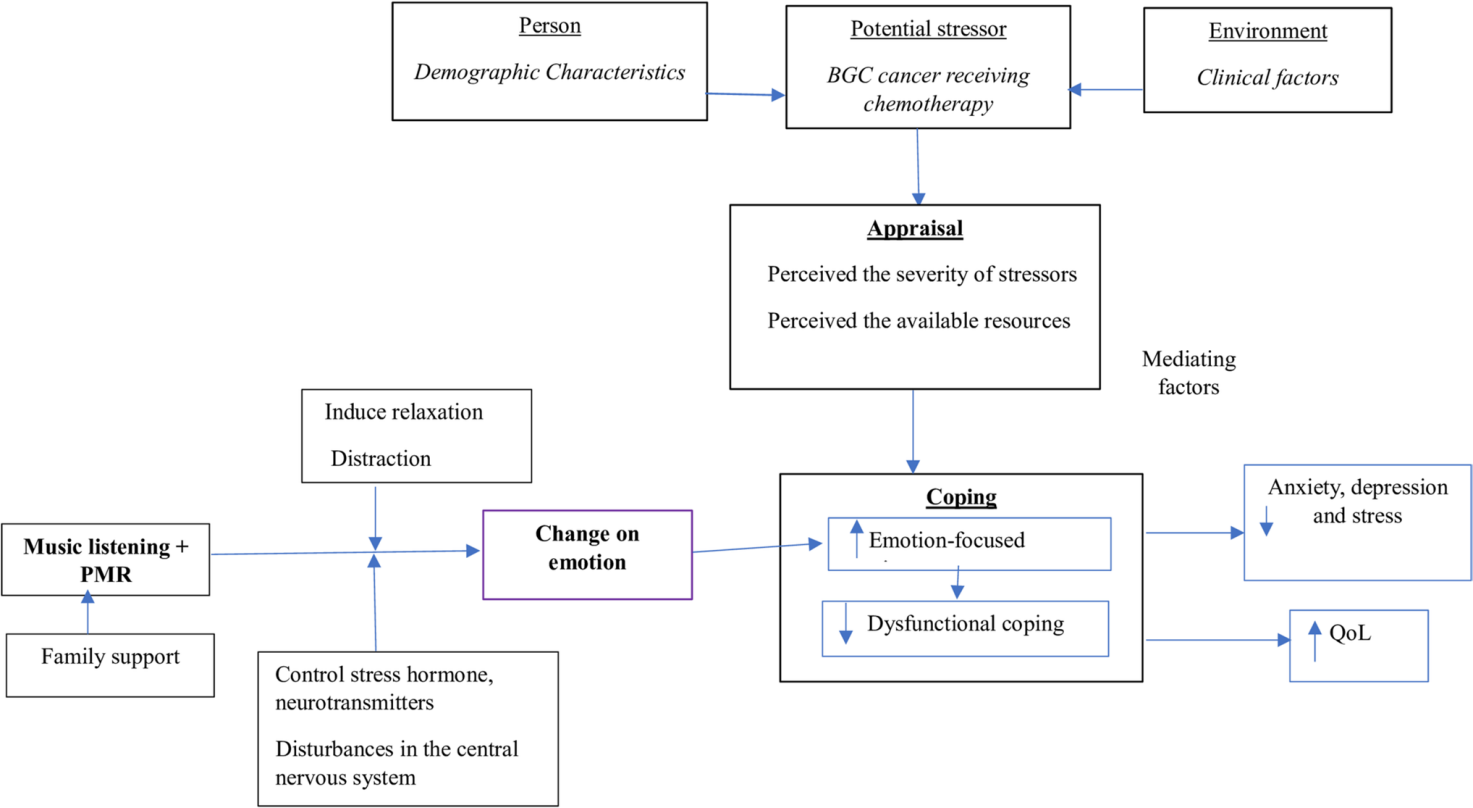
The conceptual framework depicting the mechanism of the music listening and progressive muscle relaxation

Development of the intervention was based on the transactional model of stress and coping [31], guidelines for progressive muscle relaxation [3], and the research team’s prior groundwork, including a systematic review [41], qualitative study [40], and pilot study [39] (see Fig. 1).

Each participant in the intervention group received a training programme for PML-PMR and engaged in 3 weeks of home-based self-practice. The training programme was delivered individually to participants by a research nurse who had been trained in progressive muscle relaxation and music intervention. Training took place in another private room in the same department before the participant’s chemotherapy session. Through the training programme, the participant received instruction in progressive muscle relaxation skills, was allowed to select their preferred music genre, and was assigned homework. The progressive muscle relaxation covered 16 muscle groups, from head to toe, with tensing and relaxing times of 5–7 s and 20–30 s, respectively, for each muscle group [3] (see SuppInfo 1). The research nurse demonstrated how to tense and relax each muscle group, and the participant then demonstrated the steps under the nurse’s supervision. After acquiring progressive muscle relaxation skills, the participant was asked to choose their preferred music from a pre-recorded list of music tracks, which included folk music, Buddhist or Christian music, revolutionary music, and Vietnamese bolero music, ground on the results of a previous study of the same population in Vietnam [40]. Instrumental music was chosen in this study since it had a better effect on anxiety reduction than vocal music [42]. This list comprised various selections of relaxing music with 60–80 bpm to maximise its relaxation effectiveness [8, 10, 15]. The authors discussed with a musician to select the list of tracks. The content of the intervention including music tracks, transcript of progressive muscle relaxation, and the recorded voice of the interventionist was evaluated by an academic professor, two professors in music therapy, a progressive muscle relaxation expert, a clinical psychologist, and an oncologist to ensure appropriateness. An entire intervention session involved 20 min of progressive muscle relaxation, followed by 20 min of passive music listening.

After training, the research nurse addressed each question and encouraged them to engage in daily self-practice in a private room in their home. A booklet outlining the intervention, audio recordings to guide the steps of progressive muscle relaxation, and the preferred music were sent to each participant to use in their home practice. Furthermore, the research nurse spoke with the participants’ main caregivers in person or by telephone to provide them with information about the negative mood states faced by patients with breast and gynaecological cancers, explain the intervention programme, and assign the caregivers the task of encouraging the participants to adhere to the programme. Additionally, to enhance adherence, a research assistant contacted the participants weekly by telephone to encourage self-practice, discuss any challenges, and provide solutions. A record sheet was given to the participants to document the time, period of practice, any undesirable events, and reasons for not practicing. After 3 weeks of intervention, the participants were encouraged to self-practice, but no phone call reminder was made.

The participants in the wait-list control group received usual care and weekly phone calls from the research assistant, which mainly included greetings, general inquiries, and reminders to return to the hospital for their scheduled chemotherapy sessions. After the T2 assessment, the research nurse provided these participants with a brief training programme and materials similar to those given to the intervention group.

### Outcome measures and instruments

The participants’ data, including their demographic and clinical information, were collected via a self-designed datasheet. Anxiety, depression, and stress were evaluated using the Vietnamese version of the DASS-21, which comprises seven items each to measure anxiety, depression, and stress and has demonstrated strong internal consistency [52]. Confirmatory factor analysis of the tool supports its structural validity in screening for psychological distress in cancer patients [20]. Additionally, it has shown excellent sensitivity and specificity in screening for breast cancer [2]. Quality-of-life was assessed using the Vietnamese Functional Assessment of Cancer Therapy-General (FACT-G) instrument, which covers physical function, social and family relationships, emotional well-being, and functional abilities; this instrument was translated and validated by the Functional Assessment of Chronic Illness Therapy organisation [14]. Functional emotion-focused and dysfunctional coping were measured using the Vietnamese version of the Brief-Cope Orientation to Problems Experienced Inventory (Brief-COPE), a tool widely used to evaluate coping among cancer patients; this tool was validated previously, yielding a Cronbach’s alpha of 0.85 [36].

The participants’ feedback and comments on the programme were gathered from daily self-report forms and through face-to-face individual interviews at T1 (see SuppInfo 2).

### Data collection

Data collection was conducted at T0, T1, T2, and T3. At T0, all participants were asked to complete the demographic data form, DASS-21, FACT-G, and Brief-COPE. Subsequently, the participants completed the DASS-21, FACT-G, and Brief-COPE again at T1, T2, and T3.

The participants in the intervention group recorded their self-practice daily in the self-report forms, which were returned to the administrative nurses at T1 and T2. The participants were not asked to record their daily self-practice from 6 weeks to 12 months post-intervention due to limited resources to manage adherence. Upon completion of the programme at week 3, the first author invited the participants in the intervention group to participate in individual face-to-face interviews in a private room in the hospital. Using purposeful sampling, the recruitment process was continued until reaching data saturation. The duration of each interview lasted between 30 and 40 min.

### Data analysis

The data were analysed using IBM SPSS (version 26), and all tests were two-sided with a level of significance set at 0.05. The intention-to-treat (ITT) principle was adopted in accordance with the CONSORT statement, and a generalised estimating equations (GEE) model was applied to compare the changes in each outcome at different time points with respect to the baseline. To quantify the effects of the intervention on the outcomes, Hedges’ *g* effect sizes were computed using the outcome change scores relative to the baseline. Hedges’ *g* values of 0.15, 0.40, and 0.75 can be interpreted to indicate small, medium, and large effects, respectively [9].

The qualitative data were analysed via qualitative content analysis [24], using NVivo software (version 12). All interviews were recorded and then transcribed verbatim. Two researchers independently conducted coding process to identify subcategories, categories, and themes. Any discrepant results are discussed to reach a consensus [24, 26].

## Results

### Baseline characteristics of the participants

**Fig. 2:**
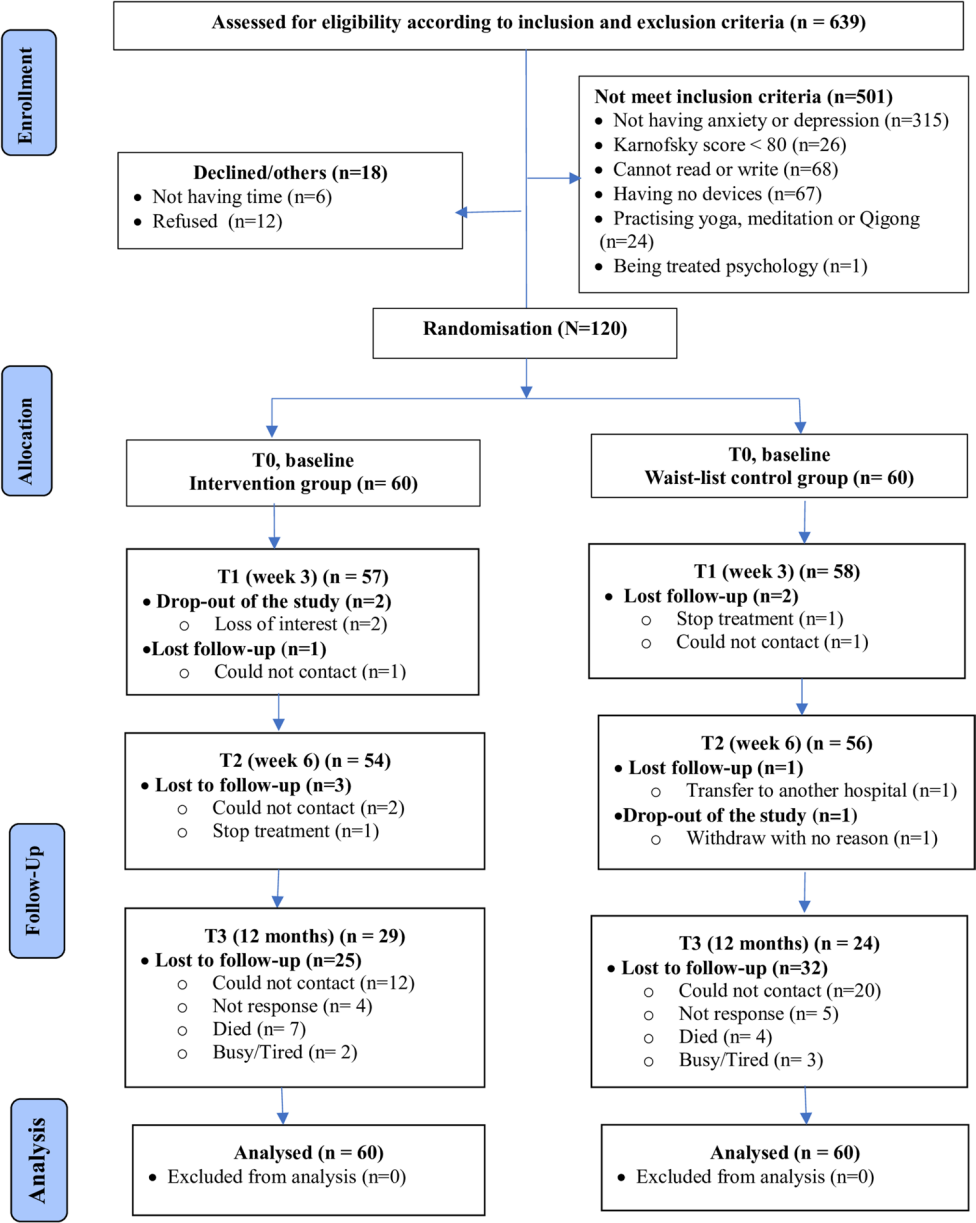
The consolidated standards of reporting trial (CONSORT) flow diagram of the study

A total of 639 patients were assessed for eligibility. Of these, 501 failed to fulfil the requirements for inclusion and 18 chose not to participate in the study. The remaining 120 participants were randomly allocated to the intervention (*n* = 60) or the wait-list control group (*n* = 60) (see Fig. 2).

The mean age of the participants was 50.1 (10.3) years. Among the participants, 48.3% possessed a secondary education or below, 62.5% were not currently employed, 52.5% resided in suburban or rural areas, and 86.3% had a monthly household income per person of less than USD 210. No significant differences in any of the demographic or clinical characteristics were found between the groups at baseline (Table 1).

**Table 1:** Participant baseline characteristics

Variables	Total (*N* = 120)			Group assignment	
				IG (*n* = 60)	WCG (*n* = 60)
**Age** (years) mean (SD)	50.08 (10.32)			53.25 (10.66)	50.92 (9.93)
**Education**					
Secondary school and lower	58 (48.3%)			29 (48.3%)	29 (48.3%)
High, vocational school	36 (30%)			17 (28.3%)	19 (31.7%)
Bachelor or higher	26 (21.7%)			14 (23.3%)	12 (20%)
**Religion**					
Non-religion	98 (81.7%)			52 (76.7%)	46 (86.7%)
Buddism	7 (5.8%)			3 (6.7%)	4 (5%)
Christian	15 (12.5%)			5 (16.7%)	10 (8.3%)
**Employment status**					
Current worker	45 (37.5%)			19 (31.7%)	26 (43.3%)
Non-current worker	75 (62.5%)			41 (68.3%)	34 (56.7%)
**Place of living**					
Urban	57 (47.5%)			29 (48.3%)	28 (46.7%)
Suburban/rural	63 (52.5%)			31 (51.7%)	32 (53.3%)
**Marital status**					
Married	96 (80%)			49 (81.7%)	47 (78.3%)
Single/divorced/separated/widowed	24 (20%)			11 (18.3%)	13 (21.7%)
**Income/month**					
> 5 million VND ($210)	86 (71.7%)			45 (75%)	41 (86.3%)
5–10 million VND	34 (28.3%)			15 (25%)	19 (31.7%)
< 10 million VND					3 (5%)
Cancer diagnosis					
Breast cancer		92 (76.7%)	50 (83.3%)		42 (70%)
Gynaecological cancer		28 (23.3%)	10 (16.7%)		18 (30%)
**Cancer stage**					
Stage 1		14 (11.7%)	7 (11.7%)		7 (11.7%)
Stage 2		34 (28.3%)	17 (28.3%)		17 (28.3%)
Stage 3		43 (35.8%)	23 (38.3%)		20 (33.3%)
Stage 4		29 (24.2%)	13 (21.7%)		16 (26.7%)
**Time from diagnosis** (months) median (IQR)		8.5 (31)	7 (31)		11 (31)
**Type of treatment**					
Chemotherapy only		93 (77.5%)	45 (75%)		48 (80%)
Chemotherapy + radiotherapy		27 (22.5%)	15 (25%)		12 (20%)
**Chemotherapy regimen**					
Single agent		69 (57.5%)	30 (50%)		39 (65%)
Combination agents		51 (42.5%)	30 (50%)		21 (35%)

*IG* intervention group, *WCG* wait-list control group, *IQR* interquartile range, *VND* Vietnam Dong,* $* USD, ^*a*^Chi-square test, ^b^Independent sample *T* test, ^c^Mann-Whitney *U* test, ^d^Fisher’s exact test

### Effects of the intervention on outcome variables

Regarding anxiety, the intervention group demonstrated significantly greater decreases in anxiety scores than the wait-list control group at both T1 (group-by-time interaction *β* = − 4.96, 95% confidence interval [CI]: − 7.96 to − 1.96, *P* < 0.001, Hedges’ *g* = 0.78) and T2 (*β* = − 5.10, 95% CI: − 8.30 to − 1.91, *P* < 0.01, *g* = 0.78), but no significant between-group difference was found at T3. Regarding depression, a significantly greater reduction was observed in the intervention group than in the control group at T2 (*β* = − 5.85, 95% CI: − 8.93 to − 2.78, *P* < 0.001, *g* = 0.81), but no significant between-group difference was observed at T1 or T3. Regarding stress, the intervention group had a significantly greater reduction in the stress score than the control group at T1 (*β* = − 4.70, 95% CI: − 7.74 to − 1.66, *P* < 0.01, *g* = 0.71), but no significant between-group difference was observed at T2 or T3.

In terms of coping, the intervention group had a greater increase in the functional emotion-focused coping score than the control group at T1, T2, and T3, although these differences were not significant. The participants in the intervention group showed a significant reduction in the dysfunctional coping score compared with the control group at T2 (*β* = − 2.53, 95% CI: − 4.29 to − 0.77, *P* = 0.01), but no significant between-group difference was observed at T1 or T3.

The intervention group had a significantly greater increase in the quality-of-life score than the control group at both T1 (*β* = 7.61, 95% CI: 2.76 to 12.46, *P* < 0.01) and T2 (*β* = 9.59, 95% CI: 3.62 to 15.57, *P* < 0.01), but no significant between-group difference was found at T3 (see Table 2).

**Table 2:** GEE model for comparing outcomes between the intervention and the wait-list control group over time

	Intervention	Control	Group effect	*P*	Time effect	*P*	Group _*_ time effect	*P*	Hedge’s g
	Mean (SD)	Mean (SD)	β (95%CI)		β (95%CI)		β (95%CI)		
Anxiety									
T0	11.44 (4.78)	11.67 (3.85)	− 1.83 (− 4.01, 0.38))	0.10					
T1	8.0 (4.57)	15.50 (6.49)			3.83 (1.55, 6.09)	< 0.001*	− 4.96 (− 7.96, − 1.96)	0.001*	0.78
T2	8.07 (4.97)	14.67 (8.39)			3.75 (1.19, 6.29)	0.004*	− 5.10 (− 8.30, − 1.91)	0.002*	0.78
T3	7.59 (8.01)	11.75 (7.34)			− 1.18 (− 4.89, 0.38)	0.53	− 2.33 (− 7.18, 0.89)	0.35	0.53
Depression									
T0	10.21 (8.52)	10.17 (5.75)	− 2.40 (− 5.67, 0.87)	0.15					
T1	7.17 (8.69)	13.92 (8.33)			2.69 (1.41, 5.25)	0.04*	− 3.18 (− 6.69, 0.32)	0.08	0.52
T2	5.79 (5.19)	13.33 (7.79)			2.98 (0.61, 5.34)	0.01*	− 5.85 (− 8.93, − 2.78)	< 0.001*	0.81
T3	7.58 (8.01)	11.75 (7.54)			− 1.42 (− 5.21, 2.38)	0.47	− 1.76 (− 7.16, 3.63)	0.52	0.53
Stress									
T0	11.24 (7.28)	11.0 (6.35)	− 2.17 (− 5.03, 0.69)	0.14					
T1	9.17 (8.15)	17.0 (7.64)			5.79 (3.75, 7.82)	< 0.001*	− 4.70 (− 7.74, − 1.66)	0.002*	0.71
T2	11.24 (7.06)	14.33 (8.60)			3.66 (1.37, 5.96)	0.002*	− 2.52 (− 5.67, 0.63)	0.12	0.53
T3	8.69 (9.06)	11.75 (7.69)			− 1.05 (− 4.86, 2.76)	0.59	− 0.89 (− 6.01, 4.23)	0.73	0.36
Functional emotion-focused coping									
T0	34.07 (6.08)	38.54 (6.79)	− 0.6 (− 2.95, 1.75)	0.62					
T1	36.38 (5.23)	37.04 (6.61)			0.89 (− 0.76, 2.54)	0.29	1.28 (− 0.93, 3.49)	0.25	0.12
T2	37.03 (5.58)	35.96 (6.26)			0.24 (− 1.59, 2.07)	0.79	1.79 (− 0.61, 4.19)	0.14	0.21
T3	37.38 (6.09)	37.62 (5.25)			1.53 (− 0.99, 4.05)	0.24	0.85 (− 2.42, 4.11)	0.61	0.04
Dysfunctional coping									
T0	17.24 (3.82)	18.54 (5.06)	− 1.42 (− 2.92, 0.09)	0.06					
T1	15.55 (3.16)	18.71 (4.54)			1.78 (0.46, 3.10)	0.01*	− 1.49(− 3.17, 0.19)	0.08	0.63
T2	15.62 (3.31)	19.83 (4.10)			2.40 (1.07, 3.73)	< 0.001*	− 2.53 (− 4.29, − 0.77)	0.01*	0.86
T3	16.41 (4.57)	18.38 (3.10)			− 0.24 (− 1.74, 1.26)	0.75	− 0.55 (− 2.99, 1.90)	0.66	0.49
Quality of life									
T0	71.84 (12.53)	77.09 (15.39)	3.49 (− 1.51, 8.49)	0.17					
T1	74.26 (14.13)	66.91 (15.36)			− 3.21 (− 6.19, 0–0.23)	0.04*	7.61 (2.76, 12.46)	< 0.01*	0.75
T2	81.99 (14.69)	71.39 (17.71)			− 2.87 (− 6.19, 0.45)	0.09	9.59 (3.62, 15.57)	< 0.01*	0.78
T3	87.97 (13.53)	78.79 (15.60)			8.68 (1.85, 15.51)	0.01*	5.68 (− 3.19, 14.54)	0.21	0.62

*T0* baseline, *T1* 3-week from T0, *T2* 6-week from T0, *T3* 12-month post-intervention. *SD* standard deviation; *Group effect*, group differences between the intervention group and the wait-list control group at the baseline (T0); *Time effect*, changes of variables in the wait-list control group at T1, T2, and T3 compared with T0; Group _*_ time effect, mean difference in changes of outcome variables in the intervention group compared with the wait-list control group at T1, T2, and T3 relative to T0; *p < 0.05

### Participants’ feedback on the intervention

The participants’ self-report forms documented that during the first week after chemotherapy, many patients reported that they could not practise daily due to fatigue and pain.

Using purposeful sampling to recruit the participants for the interviews ensured a diverse range of demographic and clinical characteristics such as age, education level, cancer stage, and chemotherapy regimen. The recruitment process was continued until reaching data saturation. A total of eleven patients participated in interviews at the hospital. The interview data were categorised into four themes (see Table 3):

**Table 3:** Themes, categories, and sub-categories of the qualitative data

Themes	Categories	Sub-categories	Example quotes
Perceived benefits of the intervention	Better psychological health	Felt comfortable Reduce anxiety Reduce stress Positive thinking	‘When I practice, I feel comfortable in my body and mind. When I focus on the exercise, I take everything out of my head (P10).’ ‘I felt less anxious, more relaxed after practising (P3).’ ‘I see a change. When a problem occurs, I have positive thinking about the problem, and I solve the issues more gently. In general, the intervention is good for mental and physical health. I really enjoyed the exercise (P2).’
	Better physical health	Better sleeping Warmer body	‘I slept very well. I could sleep continuously until morning. Before I used to wake up at midnight (P2).’ ‘I felt my body getting warmer. In the morning, I did not feel cold anymore. I used to feel cold, especially in the morning around 4–5 am. So, I needed to cover myself with a blanket, but my feet, soles of the feet and hands are still cold. However, from the day I practiced this exercise, I saw an improvement in the problem of the soles of the feet and warm hands. I find it (the intervention) very effective (P2).’
	Better family relationship		‘My family members are a lot happier and more bonded together. Thank you so much for your developing this programme (P2).’
Elements of a successful intervention	A suitable intervention design	Appropriate length of time Suitable for health status of the patients A good voice in the recorded files A perfect music collection	‘The length is suitable (P5).’ ‘It (the intervention) is appropriate for my present health situation (P9).’ ‘Your recording is very good and your voice is inspiring, putting listeners at ease and comfort (P2).’ ‘I really like the selected music. When I finished practising (PMR), it’s time to listen to the music. For the first 5–10 min, I felt like I was going to a fresh forest, bringing me back to nature… it’s very good, but to have the feeling, the listener has to really be harmonious with the music… feeling that the first 5–10 min are very flying (P3).’
	Combination of music listening and progressive muscle relaxationcreates resonance effect on relaxation	The two parts support each other	‘I think the progressive muscle relaxation part and the music listening part cannot be separated, it supports each other. The progressive muscle relaxation helps stretch the muscles to make the body circulate. The music helps me relax my brain so that I can rest after muscle exercise (P8).’
	Family support	Remind the participants to practice Encourage the participants to practice Not disturb during practicing	‘Some days I didn’t practice, and my husband reminded me that she (the PI) told me (about the need of self-practice daily) why don’t I practice (P10).’ ‘At home, whenever I feel discouraged, my husband also encourages me to try to practice, there is nothing superfluous (P4).’ ‘My husband is very kind. When I returned home, I told my children (about the programme). Everyone was supportive because this was an experimental study, so it was very serious. So, my family members were also serious while I practised at home. When I practised, everyone in my family stayed away from other places so that I could practice on my own. They kept quiet to create the best conditions for me to practice (P2).’
	Facilitators for self-practice at home	Have a quiet and private room at home Easy to use materials	‘I have my own room. It’s quiet. I just lay down and practise (P3).’ ‘I opened it then practising. It’s easy. I did not see any difficulty (P3).’
Difficulties for practice at home	Minor side effects	Cramp Pain muscles Felt tired	‘The first day I practised, I felt pain but then the pain went away (P3).’
			‘Some days I felt a bit tired, probably because I stretched my muscles so much (P4).’
Comments for the intervention	Practicing under supervision of an instructor to maximise effectiveness		‘In my opinion, in order to achieve maximum results, it is essential to provide a facility where everyone can practice and a manager and expert to support. If practicing at home, some individuals may not follow the steps correctly; (they) must have a guide (P2).’

#### Theme 1: Perceived benefits of the intervention

The participants reported multiple benefits from the intervention. In terms of mood states, the participants felt a sense of comfort, reduced anxiety and stress, increased optimism, and more positive thoughts. Physically, the participants noticed an improvement in their sleep quality and a decrease in feeling cold. Additionally, the intervention positively impacted their family relationships, with two participants expressing that their family members were happier and more connected.

#### Theme 2: Elements of a successful intervention

The participants identified key elements that contributed to the success of the intervention. They positively evaluated the intervention's length and materials, finding them suitable for their physical condition. The recorded voice and selected music enhanced their experience by creating a pleasant atmosphere. The combination of PML-PMR was highlighted as crucial for relaxation. They accordingly viewed music listening and progressive muscle relaxation as interdependent. Family support was crucial, as family members reminded the participants to practise daily and helped create a distraction-free setting. Moreover, facilitators of self-practice at home, including the ability to practice in a standard environment, and easy access to materials, were also reported as beneficial.

#### Theme 3: Difficulties in practising at home

Two patients reported that they had a cramp in the back and felt slight pain in their muscles on the first day of practice. These challenges and side effects affected their adherence to the intervention.

#### Theme 4: Comments on the intervention

One participant provided feedback suggesting that to maximise the effectiveness of the intervention, patients should practise under the guidance and supervision of an instructor. This participant expressed a concern that practising at home without guidance could cause individuals to follow the steps incorrectly.

## Discussion

Drawing on the transactional model of stress and coping, this is the first theory-driven, evidence-based randomised controlled trial of the effects of home-based PML-PMR to manage the negative mood states experienced by patients with breast and gynaecological cancers who are receiving chemotherapy. The results of the study indicate that the intervention significantly reduced the participants’ anxiety, depression, stress, and dysfunctional coping and improved their quality-of-life at short-term assessments. An improvement in emotion-focused coping was also observed, although this was not statistically significant.

The study findings demonstrate that the participants in the intervention group had significantly greater reductions in anxiety levels at T1 and T2 and in depression levels at T2 than those in the wait-list control group. The results are consistent with those of Zhou et al. [54], who showed that PML-PMR reduced anxiety and depression in female patients with breast cancer after radical mastectomy. However, the intervention tested by Zhou et al. was delivered twice a day and thus required more manpower. Moreover, their study focused only on anxiety and depression during hospitalisation, but these symptoms arise at diagnosis and remain throughout treatment and for a long period [48]. Thus, interventions should address patients’ negative mood states not only during hospitalisation but also after discharge. The intervention introduced in the current study was developed to allow patients to practise independently. It is less labour-intensive than the intervention developed by Zhou et al. [54]; feasible in any setting, including patients’ homes; and enables patients to practise consistently to relieve long-term stress.

The results also suggest that this intervention is effective and useful for reducing stress levels among cancer patients receiving chemotherapy. In other studies, PML-PMR was found to effectively reduce stress in other populations, such as intensive care nurses [43] and nursing students before exams [21]. These findings indicate the potential application of this combined approach in various settings and populations. Listening to calming music can stimulate a patient’s hypothalamus and peripheral system to reduce their cortisol level [47]. Progressive muscle relaxation can reduce muscle tension by helping individuals become more aware of their bodily sensations and promoting a sense of mindfulness [28, 29].

The current study showed a significantly greater improvement in the overall quality-of-life of participants in the intervention group than in the wait-list control group at T1 and T2. Although no previous study has assessed the impact of PML-PMR on the quality-of-life of patients with cancer, a similar significant improvement was found in previous studies on pregnant women and family caregivers [1, 16]. Passive music listening and progressive muscle relaxation have been proven to improve various aspects of physical, social/family, emotional, and functional well-being, such as nausea, vomiting, and pain [7, 51]; mood [7]; a sense of comfort [4]; and insomnia [5, 30].

More than music medicine, music therapy establishes the therapeutic relationship and the trained music therapist supports the client throughout the intervention in optimal environments. However, the number of music therapists is limited especially in developing countries such as Vietnam and the music therapy service is not covered by insurance in many countries making access to these services challenging. In this context, using passive music listening may be an effective alternative solution. To address the limitations of the home-based intervention, such as the lack of face-to-face support of the intervener, and adherence to the intervention of the participants, our study employed various strategies. Apart from the commonly used strategies including, phone calls and distributing guidelines and forms as a reminder to participants, we sought support from family members of the participants. Family members’ reminders and encouragement contributed to increased patient compliance. In addition, the family members also help pảticpants to practice in a quiet, comfortable room without being disturbed allowing them to concentrate more [3, 18]. This environment is the ideal condition to achieve the best level of relaxation by the intervention [3].

### Study limitations

This study has several limitations of note. First, due to the nature of the study, the participants and the intervener needed to be actively involved in the intervention [27]; thus, blinding them was impossible. Second, because of inadequate resources, our study design lacked a sham arm, a passive music listening arm, and a progressive muscle relaxation arm. Lastly, we provided the intervention to the participants in the wait-list control group after the assessment at T2 might introduce contamination effects during the T3 assessment. Therefore, the primary objective at T3 was to evaluate the influence of the timing of intervention delivery and the strategies provided in terms of enhancing patient adherence, rather than the long-term effects of the intervention.

### Implications for research and practice

Future study designs may include a sham group to enable the detection of a placebo effect. To achieve this, listening to music could be replaced by listening to audiobooks and reflective listening sessions [6]. A sham progressive muscle relaxation exercise could be designed by instructing participants to breathe normally and constantly perform an activity, such as tensing one muscle or moving another part of the body, instead of all 16 muscle groups in turn [22]. The findings demonstrated the effectiveness of PML-PMR in terms of improving physical and functional well-being. In addition, PML-PMR have been found to be beneficial for patients with other types of cancer and chronic diseases [17, 46]. More studies on the effects of PML-PMR on other outcomes and in different patient populations are warranted.

Regarding practice, this intervention should be considered in the context of managing negative mood states in patients. Healthcare staff will need to participate in training courses on passive music listening and progressive muscle relaxation to allow them to use the intervention in routine patient care. Regarding patients, noncompliance mainly occurs during the first week after chemotherapy, when the adverse effects cause some patients to be fatigued. To encourage patients to practise the intervention, telephone, and text message reminders should be intensified [23].

## Conclusion

The home-based PML-PMR was effective in terms of reducing participants’ anxiety and stress and improving their quality-of-life during the 3-week intervention period. The effects of the intervention on anxiety, depression, and quality-of-life were sustained at week 6. This intervention can be considered for inclusion in routine clinical practice to manage the negative mood states experienced by cancer patients.

## Supplementary Information

Below is the link to the electronic supplementary material.

Supplementary file1 (DOCX 16 KB)

Supplementary file2 (DOCX 15 KB)

## Data Availability

The dataset is not publicly available due to privacy or ethical restrictions. The data are available on request from the corresponding author.
