# Supplementary material for: Randomised controlled trial of music listening combined with progressive muscle relaxation for mood management in women receiving chemotherapy for cancer
Source: Support Care Cancer. 2025 Mar 4;33(3):245. doi: 10.1007/s00520-025-09281-4 (PMC11880154; doi:10.1007/s00520-025-09281-4)
Supplement: Supplementary file 1 — Supplementary file1 (DOCX 16 KB) [file 520_2025_9281_MOESM1_ESM.docx]

**Interview guide**

1. Could you describe the experience that you had during the intervention?
2. What did you change before and after 3 weeks of the intervention?
3. What is the role of the family member at home during the intervention?
4. What is your opinion about progressive muscle relaxation?
5. What is your opinion about the music intervention part?
6. What part of the intervention should be changed to be better?
