# Supplementary material for: Randomised controlled trial of music listening combined with progressive muscle relaxation for mood management in women receiving chemotherapy for cancer
Source: Support Care Cancer. 2025 Mar 4;33(3):245. doi: 10.1007/s00520-025-09281-4 (PMC11880154; doi:10.1007/s00520-025-09281-4)
Supplement: Supplementary file 2 — Supplementary file2 (DOCX 15 KB) [file 520_2025_9281_MOESM2_ESM.docx]

| **No.** | **Muscle** | **Tensing Instruction** |
| --- | --- | --- |
| **1** | Forehead | Lift eyebrows as high as possible |
| **2** | Eyes and nose | Closing eyes tightly and squinting and wrinkling nose at the same time. |
| **3** | Mouth and jaw | Bite hard and pull back the corners of the mouth |
| **4** | Neck | Pull the chin downward toward the chest and, at the same time, try to prevent it from actually touching the chest. |
| 5 | Dominant hand and forearm | Make tight fist |
| 6 | Dominant biceps | Pull and press the elbow inward toward the body |
| 7 | Non-dominant hand and forearm | Make tight fist |
| 8 | Non-dominant biceps | Pull and press the elbow inward toward the body |
| 9. | Chest, shoulders, and upper back | Taking a deep breath, hold it, and at the same time, pull the shoulders back and try to make the shoulder blades touch |
| 10 | Abdomen | Take a deep breath and tighten the abdomen as much as you can |
| 11. | Dominant the upper leg | Lift the leg a little bit |
| 12 | Dominant calf | Pull the toes upward toward the head |
| 13 | Dominant foot | Point the toes, turn your foot inward, and at the same time, curl the toes. (not more than 5 seconds to prevent cramps) |
| 14 | Nondominant upper leg | Lift the leg a little bit |
| 15. | Non-dominant calf | Pull the toes upward toward the head |
| 16. | Non-dominant foot | Point the toes, turn your foot inward, and at the same time, curl the toes. (not more than 5 seconds to prevent cramps) |
